# The Effect of Preoperative Biliary Drainage with or without Pancreatic Stenting on Complications after Pancreatoduodenectomy: A Retrospective Cohort Study

**DOI:** 10.1155/2021/5572395

**Published:** 2021-04-29

**Authors:** Jiangtao Chu, Shun He, Yan Ke, Xudong Liu, Peng Wang, Wei Zhang, Guotong Qiu, Chengfeng Wang, Jianwei Zhang, Guiqi Wang

**Affiliations:** ^1^The Department of Endoscopy, National Cancer Center/National clinical research center for Cancer/Cancer Hospital, Chinese Academy of Medical Sciences and Peking Union Medical College, Beijing 100021, China; ^2^The Department of Pancreatic and Gastric Surgery, National Cancer Center/National clinical research center for Cancer/Cancer Hospital, Chinese Academy of Medical Sciences and Peking Union Medical College, Beijing 100021, China

## Abstract

**Background:**

The necessity of preoperative biliary drainage (PBD) prior to pancreaticoduodenectomy (PD) is still controversial. However, in some settings, PBD with endoscopic retrograde cholangiopancreatography (ERCP) procedure is recommended as a preferred management. Meanwhile, pancreatic duct stenting in the drainage procedure is rarely performed for selected indications, and its associated complications after PD remain quite unknown.

**Methods:**

A retrospective observational longitudinal cohort study was performed on patients who underwent PBD and PD from a prospectively maintained database at the National Cancer Center from March of 2015 to July of 2019. Patients who underwent biliary stenting alone, biliary and pancreatic stenting, were distributed into two study cohort groups, and their records were scrutinized for the incidence of postoperative complications.

**Results:**

A total of 83 patients who underwent successful PD after biliary drainage were identified. 29 patients underwent nasobiliary drainage (ENBD)/plastic or metal bile duct stenting (BS) and pancreatic duct stenting (PS group), and 54 patients underwent only ENBD/BS, without pancreatic duct stenting (NPS group). No differences were found between the two groups with respect to in-hospital time, overall complication rate, respective rate of serious (grade 3 or higher) complication rate, bile anastomotic leakage, bleeding, abdominal infection, surgical wound infection, organ dysfunction, and pancreatic anastomotic leakage. Postoperative gastrointestinal dysfunction rates differed significantly, which occurred in 3 (5.56%) cases in the NPS group, compared with 6 (20.7%) cases in the PS group (*P* = 0.06). In the univariate and multivariate regression model analysis, pancreatic duct stenting was correlated with higher rates of gastrointestinal dysfunction [odds ratio (OR) = 4.25, *P* = 0.0472].

**Conclusion:**

Our data suggested that PBD and pancreatic duct stenting prior to pancreatoduodenectomy would increase the risk of postoperative delayed gastric emptying, while the overall incidence of postoperative complications and other complications, such as pancreatic leakage and bile duct leakage, showed no statistical difference.

## 1. Introduction

Biliary obstruction is a common problem in patients undergoing pancreaticoduodenectomy (PD). However, the necessity for preoperative biliary drainage (PBD) remains controversial [1]. A recent well-published study suggests that routine PBD is not recommended because it is associated with increased complications when PD is conducted [2]. However, if the bilirubin is markedly elevated in the symptomatic patient, or surgery needs to be delayed to optimize medical comorbidities or to manage neoadjuvant therapy, PBD is still required [1]. Transpupillary endoscopic biliary drainage (EBD) with endoscopic retrograde cholangiopancreatography (ERCP) is recognized as the gold standard procedure for malignant biliary obstruction (MBO) prior to PD [3].

However, the ERCP procedure can cause complications such as pancreatitis, cholangitis, bleeding, and perforation, which delays the subsequent pancreaticoduodenectomy, and may cause postoperative complication [4].

Postoperative pancreatitis is a common complication after ERCP, especially for patients with difficult cannulation. Many studies have reported that pancreatic duct stenting can reduce the occurrence of pancreatitis after ERCP [5]. A cost-effective analysis showed that restricting preventive pancreatic stenting to high-risk patients is the most cost-effective strategy [6]. The European Society of Gastrointestinal Endoscopy strongly recommends that patients at high risk of postoperative pancreatitis, such as inadvertent guidewire insertion, opacification of the pancreatic duct, double-guidewire cannulation, could select pancreatic stenting [7].

Therefore, the correlation between pancreatic duct stenting and its resultant complications after pancreaticoduodenectomy needs to be evaluated. The purpose of our study was to analyze and assess the effect of pancreatic duct stenting on the complication of pancreaticoduodenectomy. This study compared the complication incidence of pancreaticoduodenectomy in patients with PBD and plastic pancreatic duct stenting (PS group) and patients with PBD alone (NPS group).

## 2. Methods

### 2.1. Patients Selection

From March 2015 to July 2019, the data of patients who underwent successful PD after biliary drainage with or without pancreatic stenting were extracted from the National Cancer Center database, and a retrospective observational cohort study was conducted. This study was approved by the institutional review board and ethics committee. Patients enrolled in the study met all the following requirements: (1) age ≧ 18 years old, (2) with low malignant biliary obstruction, (3) successful PBD, or with/without pancreatic duct stenting, and (4) successful pancreaticoduodenectomy. Those who have undergone short-circuit surgery, percutaneous biliary drainage, or intraoperative tumor metastasis or unable to undergo radical resection were excluded.

A total of 83 patients who underwent successful PD after biliary drainage were identified. 31 patients had periampullary carcinoma, 16 had a tumor located in the head of the pancreas, 22 had cholangiocarcinoma, and 14 had duodenal adenocarcinomas, respectively. Among the 83 who had successful PD, 29 patients underwent biliary drainage and pancreatic duct stenting (PS group), and 54 patients underwent only biliary drainage without pancreatic duct stenting (NPS group).

### 2.2. Biliary Drainage and Pancreaticoduodenectomy

PBD procedure was performed with nasobiliary drainage, metal stent, or plastic stenting. When the double-guidewire method was selected for difficult bile duct canulation or the guidewire was accidentally inserted into the pancreatic duct, a 5-Fr plastic pancreatic duct stent with flanks on the inside was routinely implanted.

The standard surgical procedure for resectable tumors was the pylorus-preserving pancreaticoduodenectomy, including removal of all lymph nodes on the right side of the portal vein and mesenteric artery [8]. If metastasis into the proximal duodenum or pylorus was suspected, a classic Whipple procedure was performed, with resection of the distal stomach. If limited metastasis into the portal or superior mesenteric vein was found, a wedge resection of these vessels was included in the procedure [8, 9]. No specific modifications of the surgical technique were expected for patients with jaundice, particularly with respect to creating the hepaticojejunostomy.

### 2.3. Data Collection and Definition

Demographic characteristics of the included patients were evaluated, including pancreaticoduodenectomy, operation time, intraoperative blood transfusion, perioperative complications, clinical pathological results, margin conditions, and other indicators. The length of hospital stay was defined from the day of surgery to the day of discharge.

The severity of perioperative complications (i.e., perioperative bleeding, abdominal infection, delayed gastric emptying, bile anastomotic leakage, and pancreatic anastomotic leakage) was divided into 5 grades as described in Table 1 [10, 11]. Grade 1-2 was defined as the mild complication, and grade 3 or higher was defined as the severe complication.

### 2.4. Primary Outcome

The main outcome measure is the incidence of complications after pancreaticoduodenectomy of the enrolled patients.

### 2.5. Secondary Outcome

The secondary outcome included hospital stay time, operative time, and intraoperative blood transfusion volume.

### 2.6. Statistical Analysis

Continuous variables were described by median and range, while categorical variables were described by frequency and percentage. To analyze the differences between the two groups (pancreatic duct stent group and nonpancreatic duct stent group), categorical variables were analyzed using Fisher's exact test, and continuous variables were tested using ANOVA. The length of hospital stay and intraoperative blood transfusion was transformed into a normal distribution through logarithmic transformation, and ANOVA test was performed. R language was used to analyze the risk factors of surgical complications in univariate and multivariate analysis. Factors with a *P* value of <0.20 in univariate analysis were included in the multivariate analysis. Patients with missing values were excluded from the analysis.

## 3. Results

Between the two groups (Table 2), there have been no statistically significant differences in all demographic data including hospital stay time, operative time, and intraoperative blood transfusion volume, except the tumor location.

The overall complication rate of the study was 68.7%, including perioperative complications, severity of (grade 3 or higher) complications, delayed gastric emptying, biliary anastomotic leakage, pancreatic anastomotic leakage, abdominal infection, and wound infection as listed in Table 3. No significant differences were found between the two groups in overall complications. While postoperative gastrointestinal dysfunction rates differed with delayed gastric emptying occurring in 3 (5.56%) in the NPS group, compared to 6 (20.7%) in the PS group (NPS vs. PS *P* = 0.06).

In the univariate regression model analysis about postoperative gastrointestinal dysfunction, pancreatic duct stenting was correlated with higher rates of gastrointestinal dysfunction [odds ratio (OR) = 4.25, *P* = 0.0472]. In the multivariate regression model, PS had more delayed gastric emptying complication than the NPS group when adjusting for age.  Table 4 shows that no other covariate was found significantly associated with the complications.

## 4. Discussion

The necessity of preoperative biliary drainage (PBD) prior to pancreaticoduodenectomy (PD) remained unclear because some studies showed that PBD procedure may be related to the complications of PD [4, 10, 12–16]. However, the PBD would be the recommended bridge management to resolve jaundice in some situations, such as acute cholangitis, obstruction with bilirubin levels exceeding 250 *μ*mol/L, severe pruritus, jaundice associated with renal failure or comorbidities, or need of neoadjuvant chemotherapy for borderline resectable lesions [3]. Meanwhile, ERCP procedure with biliary stent or nasobiliary tube for PBD was preferred because of higher safety and better oncological outcomes [17, 18].

Postoperative pancreatitis was the most common complication of ERCP. According to reports, the incidence of postoperative pancreatitis was 2-10% for all patients, 2-4% for low-risk groups, and 8-40% for high-risk groups [19]. Pancreatic duct stent implantation was recommended to prevent postoperative pancreatitis [20], especially when the guidewire was accidentally inserted into the pancreatic duct, or the double-guidewire method was adopted.

There were few studies to evaluate the influence of pancreatic duct stenting during PBD on the complications of pancreaticoduodenectomy. A retrospective study by John et al. in 2018 showed that simultaneous cholangiopancreatic duct drainage increased the incidence of postoperative pancreatic leakage [21]. This study enrolled only 5 cases of simultaneous cholangiopancreatic duct drainage, which was regarded as a rare event during data analysis and Firth logistic regression. An opposite result was presented in our study, showing no significant difference between the two groups regarding the incidence of postoperative pancreatic leakage or bile duct leakage. The different results may be contributed to the method of pancreaticointestinal anastomosis and the proficiency of pancreatic duct stenting.

When analyzing each postoperative complication separately, there has been no statistical difference in terms of operation time, hospital stay, intraoperative blood transfusion, postoperative bleeding, pancreatic leakage, bile leakage, wound infection, abdominal infection, and organ dysfunction between the two groups. However, the incidence of delayed gastric emptying in the pancreatic duct stenting group was higher than that in the NPS group (*P* = 0.06). The multivariate and univariate adjusted analysis showed that pancreatic duct stenting was an independent risk factor for delayed gastric emptying (OR = 4.25, *P* = 0.0472), which was considered as one of the most common complications after pancreatic surgery, with about 19%-57% incidence [22].

The potential mechanisms for delayed gastric emptying after PD have been unclear. Some studies have reported that other postoperative complications, such as pancreatic leakage, accumulation of peripancreatic fluid, or abdominal abscess, could increase the incidence of gastric emptying disorders [23]. However, many patients develop delayed gastric emptying in the absence of pancreatic leakage or other abdominal infections, which may represent a distinct pathology [24]. The study is the first to report that the PBD and pancreatic duct stenting prior to pancreatoduodenectomy would increase the risk of postoperative delayed gastric emptying. The potential pancreatic inflammation after pancreatic duct stenting may play an important role in delayed gastric emptying. To most patients, although delayed gastric emptying was generally not life-threatened, patients could suffer abdominal discomfort, longer hospitalization time, increased hospitalization costs, and reduction of postoperative life quality.

The results suggest a hard choice in the PBD procedure when considering post-ERCP pancreatitis and outcomes of pancreaticoduodenectomy. Therefore, a PBD procedure without influence on the pancreatic duct would be preferred, such as endoscopic ultrasonography- (EUS-) guided biliary drainage method which can avoid contacting the pancreatic bile duct segmental in accessing the bile duct [25].

Several limitations in our study cannot be neglected. First, pancreaticoduodenectomy was not performed by the same surgeon. The difference in surgery proficiency may be one uncertainty, which affected the incidence of postoperative delayed gastric emptying. Second, our study did not find a significant difference in the incidence of other complications between the two groups, which may be attributed to the small number of cases of preoperative simultaneous cholangiopancreatic duct drainage. We cannot exactly exclude the possibility that pancreatic duct stent implantation may increase the incidence of other complications, such as surgical wound infection and abdominal infection. Also, the increase of the incidence of delayed gastric emptying needs to be clarified by a multicenter study.

## 5. Conclusion

This study has shown that preoperative biliary drainage and pancreatic duct stenting prior to pancreaticoduodenectomy would increase the risk of postoperative delayed gastric emptying, while with no significant difference to the overall incidence of postoperative complications. More studies, especially RCTs, are preferred to verify the results in the future study.

## Figures and Tables

**Table 1:** Complication criteria.

Grade definition
0: no complications
1: Oral medications or supportive care
2: IV medical therapy with resolution or antibiotics or specialized nutritional support
3: interventional radiology, endoscopic, or operative intervention
4: chronic deficit or disability associated with sequelae of this event
5: death associated with sequelae of this event

**Table 2:** Demographic and clinical characteristics of patients NPS group and PS group.

	[ALL]	NPS^∗^	PS^∗^	*P* value
	*N* = 83	*N* = 54	*N* = 29	
Sex (female)	42 (50.6%)	24 (44.4%)	18 (62.1%)	0.193
Age	58.0 [51.5; 63.0]	57.0 [51.0; 62.8]	59.0 [53.0; 63.0]	0.436
^#^BMI				0.684
Fat	35 (42.2%)	24 (44.4%)	11 (37.9%)	
Low	4 (4.82%)	2 (3.70%)	2 (6.90%)	
Normal	44 (53.0%)	28 (51.9%)	16 (55.2%)	
Biliary drainage				1.000
ENBD	66 (79.5%)	43 (79.6%)	23 (79.3%)	
Metal biliary stent	1 (1.20%)	1 (1.85%)	0 (0.00%)	
Plastic bile duct stent	16 (19.3%)	10 (18.5%)	6 (20.7%)	
Tumor location				0.024
Ampulla	31 (37.3%)	26 (48.1%)	5 (17.2%)	
Bile duct	22 (26.5%)	12 (22.2%)	10 (34.5%)	
Duodenum	14 (16.9%)	9 (16.7%)	5 (17.2%)	
Pancreas	16 (19.3%)	7 (13.0%)	9 (31.0%)	
Stay time	18.0 [14.0; 22.5]	20.0 [14.0; 23.0]	17.0 [15.0; 22.0]	0.681
Blood transfusion	300 [0.00; 750]	0.00 [0.00; 675]	500 [0.00; 800]	0.070
Operation duration	366 [306; 454]	359 [314; 466]	369 [300; 432]	0.909

**Table 3:** Outcomes of patients' PS group and NPS group.

	[all]	NPS^∗^	PS^∗^	*P* value
	*N* = 83	*N* = 54	*N* = 29	
Complication criteria				0.57
None	26 (31.3%)	15 (27.8%)	11 (37.9%)	
Mild	42 (50.6%)	28 (51.9%)	14 (48.3%)	
Severe	15 (18.1%)	11 (20.4%)	4 (13.8%)	
Bile anastomotic leakage	7 (8.43%)	4 (7.41%)	3 (10.3%)	0.691
Bleeding				0.846
Blood transfusion	11 (13.3%)	8 (14.8%)	3 (10.3%)	
Dead	2 (2.41%)	2 (3.70%)	0 (0.00%)	
Hemostasis	3 (3.61%)	2 (3.70%)	1 (3.45%)	
None	67 (80.7%)	42 (77.8%)	25 (86.2%)	
Abdominal infection				0.670
Interventional therapy	10 (12.0%)	8 (14.8%)	2 (6.90%)	
Medication	24 (28.9%)	15 (27.8%)	9 (31.0%)	
None	49 (59.0%)	31 (57.4%)	18 (62.1%)	
Surgical wound infection	13 (15.7%)	8 (14.8%)	5 (17.2%)	0.761
Gastrointestinal dysfunction	9 (10.8%)	3 (5.56%)	6 (20.7%)	0.060
Organ dysfunction	6 (7.23%)	5 (9.26%)	1 (3.45%)	0.660
Pancreatic anastomotic leakage				0.380
None	58 (69.9%)	36 (66.7%)	22 (75.9%)	
A	8 (9.64%)	7 (13.0%)	1 (3.45%)	
B	17 (20.5%)	11 (20.4%)	6 (20.7%)	

**Table 4:** The univariate and multivariate analysis of predictive factors for postoperative delayed gastric emptying.

	[ALL]	Univariate analysis		Multivariate analysis	
	*N* = 83	OR	*P* value	OR	*P* value
Sex (male)	41 (49.4%)	2.16 [0.51;11.5]	0.313		
Age	58.0 [51.5; 63.0]	1.07 [0.98; 1.18]	0.085	1.07 [0.97; 1.17]	0.146
BMI			0.310		
Fat	35 (42.2%)	Ref.			
Low	4 (4.82%)	NA			
Normal	44 (53.0%)	NA			
Stay time	18.0 [14.0; 22.5]	0.99 [0.93; 1.06]	0.924		
Tumor location			0.522		
Ampulla	31 (37.3%)	Ref.			
Bile duct	22 (26.5%)	3.05 [0.51; 26.7]			
Duodenum	14 (16.9%)	2.36 [0.22; 25.0]			
Pancreas	16 (19.3%)	1.02 [0.03; 13.6]			
Blood transfusion	414 (490)	1.00 [1.00; 1.00]	0.145		
Operation duration	366 [306; 454]	1.00 [1.00; 1.01]	0.524		
Biliary drainage			1.000		
ENBD	66 (79.5%)	Ref.			
Metal biliary stent	1 (1.20%)	NA			
Plastic bile duct stent	16 (19.3%)	NA			
PDS	29 (34.9%)	4.25 [0.99; 22.9]	0.0472	4.36 [0.97; 19.42]	0.053
Bile leakage	7 (8.43%)		1.000		
Bleeding			0.636		
Blood transfusion	11 (13.3%)	Ref.			
Dead	2 (2.41%)	NA			
Hemostasis	3 (3.61%)	NA			
None	67 (80.7%)	NA			
Abdominal infection			1.000		
Interventional therapy	10 (12.0%)	Ref.			
Medication	24 (28.9%)	0.78 [0.06; 26.7]			
None	49 (59.0%)	1.14 [0.16; 32.2]			
Surgical wound infection	13 (15.7%)	0.72 [0.03; 4.63]	1.000		
Organ dysfunction	6 (7.23%)		1.000		
Pancreatic leakage			0.866		
0	58 (69.9%)	Ref.			
A	8 (9.64%)	NA			
B	17 (20.5%)	NA			

## Data Availability

From March 2015 to July 2019, the data of patients who underwent successful PD after biliary drainage with or without pancreatic stenting were extracted from the National Cancer Center database.
